# Editorial: Early life programming in poultry: Recent insights and interventional approaches

**DOI:** 10.3389/fvets.2022.1105653

**Published:** 2023-01-05

**Authors:** Abdel-Moneim Eid Abdel-Moneim, Abdelrazeq M. Shehata, Vinod Kumar Paswan

**Affiliations:** ^1^Department of Biological Applications, Nuclear Research Center, Egyptian Atomic Energy Authority, Cairo, Egypt; ^2^Department of Animal Production, Faculty of Agriculture, Al-Azhar University, Cairo, Egypt; ^3^Department of Dairy Science and Food Technology, Institute of Agricultural Sciences, Banaras Hindu University, Varanasi, India

**Keywords:** early-life programming, in-ovo feeding, early nutrition, environmental manipulation, gut health, immune system, antibiotic alternatives, poultry

“Early-life programming” describes how exposure to certain conditions during embryonic development or the early post-hatching period can change the normal development process, permanently altering how a bird's body looks and functions (1–4). Early-life programming can affect a bird for the rest of its life, altering its growth performance, tolerance to harsh environmental conditions, resistance to diseases like enteric infection and inflammation, immune function, metabolic disorders, and overall production(2–4). Considerable evidence suggests that early-life programming can alter the phenotype and performance of chicks in significant ways by modifying the expression of specific genes. In both the pre- and post-hatching phases, these treatments alter the environment and the diet of the organisms involved (1, 2).

This Research Topic is aimed at collecting papers suitable for improving our understanding of early life programming and its long-term role in minimizing environmental and health challenges. Its goal is also to share our knowledge on nutritional and environmental factors involved in early-life programming in poultry, such as in-ovo feeding, inadequate conditions, nutrient deficiencies, and sexing methods.

In this special e-book, there are nine papers covering the above-mentioned aspects. Three papers out of nine (33.3%) discuss the role of in-ovo feeding on growth performance, antioxidative capacity, breast development, glycogen reserves, nutrient absorption, immunity, and disease resistance. The excessive use of antibiotics in commercial poultry farms results in multidrug resistance and hinders the efficiency of antibiotics, causing increased threats to human and animal health. Therefore, the use of some natural bioactive molecules that can enhance the immune system, growth performance, and health status can be a promising alternative to antibiotics (5–7). A study by Bhanja et al. shows how silver nanoparticles alone or in combination with some nutrients play an important role in inducing innate or adaptive immune responses in broilers. They demonstrated that in-ovo supplementation of silver nanoparticles with amino acids, vitamins, and trace elements improved post-hatch growth and immune responses in broilers and in particular that 50 μg/egg silver nanoparticles, in combination with vitamins (B1 and B6) and trace elements (Zn and Se), improved growth performance, and that 50 μg/egg silver nanoparticles with trace elements and amino acids enhanced immune responses in broilers challenged with Newcastle disease virus.

Given the biological link between methionine and/or disaccharide and post-hatching growth and development, Dang et al. investigated the effects of in-ovo feeding with methionine (Met) and/or disaccharide (DS) on the breast muscle and small intestine of the goose, together with the contents of its glycogen stores, the activities of its digestive enzymes, and levels of antioxidants in the jejunum. They found that DS injections enhance glycogen reserves and control muscle growth-related gene expression, increasing breast muscle metrics and hatchling weight. Delivering DS into the embryo improved digestive enzyme activities, nutrient transport enzyme activities, jejunal villus indices, and regulated nutrient transport-related gene expression, increasing post-hatch nutritional absorption. Met treatment in-ovo improved breast muscle metrics by regulating growth-related gene expression. Furthermore, Met treatment temporarily improved digestive enzymes, nutrient transport enzymes, small intestine parameters, and nutrient transport-related gene expression, and constantly improved jejunal villus parameters and jejunal antioxidant capacity status, which supported nutrient absorption post-hatching. As a result, they concluded that in-ovo administration of DS with Met is an effective method for enhancing the goslings' post-hatching nutrient absorption and breast muscle growth.

Heat stress promotes oxidative stress, which alters enzyme functions and immunological responses in broilers. Han et al. examined the effects of L-Leucine in-ovo feeding on broiler growth, organ weight, serum metabolites, antioxidant indices, and gene expression during chronic heat stress. Overall, they concluded that L-Leucine in-ovo feeding mitigates oxidative damage and improves antioxidant capacity in broiler chickens exposed to heat stress.

The burden of pathogens can be decreased by the use of probiotics because they initiate resistance to bacterial colonization and boost mucosal immunity (1, 2, 8–10). Yu et al. investigated how consumption of *Bacillus coagulans* (*B. coagulans*) and *Lactobacillus plantarum* (*L. plantarum*) affected broilers when they were exposed to *Escherichia coli* lipopolysaccharide (LPS). They demonstrated that supplementing broiler chickens with *B. coagulans* and *L. plantarum* enhanced their growth performance, immunity, and antioxidant ability, and reduced the LPS-induced inflammatory response *via* modulating the gut microbiota.

Exogenous emulsifiers improve lipid utilization and enhance nutrient absorption in broilers. The purpose of the research conducted by Li, Abdel-Moneim, Mesalam et al. was to investigate the genes that are crucial to the regulatory impact of lysoforte. They reported that at least 29 genes (including REG4, GJB1, KAT2A, APOA5, SERPINE2, ELOVL1, ABCC2, ANKRD9, CYP4V2, and PISD) and several signaling pathways may be involved in enhancing jejuna morphology in broiler birds. These findings clarified the role of LFT in maintaining the health and integrity of the intestines in broiler chickens.

The effects of hypoxia exposure (HE) on the embryogenesis of chickens are undesirable; however, the mechanism underlying the responses of the heart to HE during embryogenesis in birds is not yet fully understood. The research of Li, Abdel-Moneim, Hu et al. aimed to identify the hub genes as well as the signaling pathways that are associated with chronic hypoxic stress. Numerous genes, including SGCD, DHRS9, HELQ, MCMDC2, and ESCO2, along with multiple signaling pathways (including MAPK, PPAR, insulin, ERI, and adrenergic signaling pathways), were revealed to potentially contribute to the heart's response to HE in chickens.

The research article by Li, Shi et al. identified the key genes involved in LPC regulation in the jejunum of birds. Many genes may be involved in influencing the jejuna morphology of birds, including RSAD2, OASL, EPSTI1, CMPK2, IFIH1, IFIT5, USP18, MX1, and STAT1.

The substantial roles that methionine (Met) plays in the synthesis of proteins and the methylation process of DNA, and its role as a precursor in the production of cysteine, glutathione, and taurine, point to the crucial nature of this amino acid. Liu et al. looked at how a methionine (Met) deficiency during the chicks' early growth (0–6 weeks) affected their performance, egg quality, and serum amino acid metabolism later in life. The growth performance, blood amino acid content, intestinal maturation, and gut microbiota of layer hens were all negatively affected by a lack of Met during the evaluation period (0–6 weeks). Met deficit during this stage led to a serum amino acid imbalance, which impacted growth performance, and had a negative effect on the development and productivity of egg-laying hens throughout the duration of the research (from week 7 to 24).

Because turkey farms often use two distinct lines: a heavy line, which is composed of males, and a laying line, which supplies dams, early sex determination is an extremely important factor in the industry. Pardo et al. demonstrated that combining egg external traits, down feather colors, and two behavioral procedures (“English method” and “slap technique”) permit successful sexing in newly hatched Andalusian turkey poults, especially for the two roan varieties (black roan and bronze roan).

Thus, this Research Topic presents an overview of existing knowledge, highlights new insights on early-life programming in poultry and draws attention to some useful applications during the perinatal period in poultry.

## Author contributions

All authors listed have made a substantial, direct, and intellectual contribution to the work and approved it for publication.
